# Antidepressant-Like Effects of Fractions Prepared from Danzhi-Xiaoyao-San Decoction in Rats with Chronic Unpredictable Mild Stress: Effects on Hypothalamic-Pituitary-Adrenal Axis, Arginine Vasopressin, and Neurotransmitters

**DOI:** 10.1155/2016/6784689

**Published:** 2016-06-20

**Authors:** Li-Li Wu, Yan Liu, Can Yan, Yi Pan, Jun-Fang Su, Wei-Kang Wu

**Affiliations:** ^1^Department of Basic Theory of Chinese Medicine, School of Preclinical Medicine, Guangzhou University of Chinese Medicine, 232 Waihuandong Road, Higher Education Mega Center, Panyu District, Guangzhou 510060, China; ^2^Department of Pathophysiology, School of Preclinical Medicine, Guangzhou University of Chinese Medicine, 232 Waihuandong Road, Higher Education Mega Center, Panyu District, Guangzhou 510060, China; ^3^Department of Pathophysiology, School of Preclinical Medicine, Sun Yat-Sen University, 58 Zhongshan Er Road, Guangzhou 510080, China

## Abstract

The aim of the present study was to investigate the antidepressant-like effects of two fractions, including petroleum ether soluble fraction (Fraction A, FA) and water-EtOH soluble fraction (Fraction B, FB) prepared from the Danzhi-xiaoyao-san (DZXYS) by using chronic unpredictable mild stress-induced depressive rat model. The results indicated that DZXYS could ameliorate the depression-like behavior in chronic stress model of rats. The inhibition of hyperactivity of HPA axis and the modulation of monoamine and amino acid neurotransmitters in the hippocampus may be the important mechanisms underlying the action of DZXYS antidepressant-like effect in chronically stressed rats.

## 1. Introduction

Depression is the commonest psychiatric disorder with such main clinical features as significant and lasting depression, mental retardation, cognitive impairment, and depression and somatic symptoms. Furthermore, depression is a risk factor for the onset of type 2 diabetes mellitus, hypertension, coronary heart disease, cancer, and so on [1], which is projected to become the second biggest contributor to the global burden of disease and disability by 2020 according to WHO's prediction.

Monoamine neurotransmitters disorder and hypothalamic-pituitary-adrenal (HPA) axis hyperactivity, widely recognized, are the two important mechanisms of the occurrence of depression. At present, based on the hypothesis of monoamine neurotransmitters function deficiency, antidepression drugs, such as the tricyclic antidepressants (TCAs), the selective serotonin reuptake inhibitors (SSRIs), the selective norepinephrine reuptake inhibitors (SNRIs), the selective norepinephrine-dopamine reuptake inhibitors, and the monoamine oxidase inhibitors (MAOIs), have been developed, which are mainly by inhibiting the transporter function of 5-hydroxytryptamine (5-HT), dopamine (DA), and norepinephrine (NE) to block the reuptake of monoamine neurotransmitters to increase the concentration of those neurotransmitters mentioned above in synaptic cleft to improve the symptoms of depression. But now, the efficacy of those drugs has been faced with serious challenges [2]. It is noted that inhibition of HPA axis activity is a very effective antidepressant treatment, but so far new antidepression drugs taking the HPA axis as the target have not been successfully tested by the clinical trials [3]. Therefore, the need for more effective and safer antidepression drugs continues to command attention in neuropsychopharmacology within the psychotherapeutic community.

Nowadays, traditional Chinese medicine (TCM) has offered prospective alternative therapy for the treatment of depression. The records of treating mental disorders could be found in ancient medicinal books, and herbal decoction Danzhi-xiaoyao-san (DZXYS), also called Jiawei-xiaoyao-san, is one of the most popular classical ones. The first description of DZXYS is recorded in* Jiaozhu Furen Liangfang “Edited and Commented Effective Formulae for Woman”* written by Xue Yi (1487–1559) in Ming Dynasty. This herbal formula is composed of the following herbs: Radix Bupleuri, Paeoniae Radix Alba,* Angelica sinensis*, bighead atractylodes rhizome,* Poria cocos*, bark of tree peony root, Fructus Gardeniae,* Mentha haplocalyx*, and Licorice. It is well known that DZXYS decoction plays an important role in the clinical therapy of depression-related disorders in China [4, 5]. In Chinese medicinal theory, DZXYS decoction exhibits therapeutic effects by releasing constraint and encouraging the free-flow of liver qi, clearing liver heat, nourishing blood, and invigorating spleen. Although this decoction has been used frequently, thus far, it remains unclear as to which active fraction of the DZXYS is responsible for the antidepressant effect and its possible regulatory mechanism.

The aim of the present study was to investigate the antidepressant-like effects of two fractions, including petroleum ether soluble fraction (Fraction A, FA) and water-EtOH soluble fraction (Fraction B, FB) prepared from the DZXYS by using chronic unpredictable mild stress- (CUMS-) induced depressive rat model. The underlying mechanism of antidepression was mainly explored by measuring the function of HPA axis and the contents of monoamine and amino acid neurotransmitters in hippocampus. In addition, as a neuropeptide, the arginine vasopressin (AVP) contents in plasma and hypothalamus were detected in the study in order to further explore the antidepressant mechanisms of DZXYS in modulating HPA axis.

## 2. Materials and Methods

### 2.1. Plant Materials

Medical plants (dried crude herbs) used for preparation of DZXYS were purchased from Medicinal Materials Company of Guangzhou, Guangzhou, China, and authenticated by Professor Ming-Ping Liu, Chinese Materia Medica College, Guangzhou University of Chinese Medicine. DZXYS consists of nine dried crude herbs listed in Table 1. The ratio of Radix Bupleuri, Paeoniae Radix Alba,* Angelica sinensis*, bighead atractylodes rhizome,* Poria cocos*, bark of tree peony root, Fructus Gardeniae,* Mentha haplocalyx*, and Licorice is 2 : 2 : 2 : 2 : 2 : 2 : 2 : 1 : 1. The voucher specimens were conserved at the herbal herbarium of Chinese Materia Medica College, Guangzhou University of Chinese Medicine, Guangzhou, China. The voucher specimens of the herbal materials were deposited for possible future comparison.

### 2.2. Preparation of Fractions from DZXYS

The crude powdered mixture of DZXYS, 5000 g, according to the prescription proportion, was added with petroleum ether (bp 60–90°C) and then water-bath-heated and refluxed for 4 times (2 h, 1.5 h, 1.5 h, and 1.5 h); the petroleum-soluble fraction (Fraction A, FA, yield: 1.24%) and precipitate were obtained. The precipitate was extracted for 3 times (2 h, 1.5 h, and 1.5 h) with boiling water, and then the combined water extracts were filtered and concentrated to 2500 mL solution which was concentrated under reduced pressure and fractionated by the addition of 1710 mL of 95% ethyl alcohol (EtOH) to give a water-EtOH soluble supernatant fraction (Fraction B, FB, the yield: 25.9%). FA and FB were lyophilized into dry powders. In this paper, the doses of FA and FB were expressed in terms of dried weight of herbal material used for extraction per unit body weight of experimental rats (g/kg).

### 2.3. Drugs, Reagents, and Chemicals

Imipramine hydrochloride (IMI) was purchased from Sigma (St. Louis, MO, USA). The assay kits of corticosterone (CORT), corticotrophin-releasing hormone (CRH), adrenocorticotropin (ACTH), and arginine vasopressin (AVP) were purchased from Nanjing Jiancheng Institute of Biotechnology, Nanjing, China. The standard 5-hydroxytryptamine (5-HT), dopamine (DA), and norepinephrine (NE) were purchased from Sigma (St. Louis, MO, USA). Disodium ethylenediaminetetraacetate (EDTA) and the standard amino acids including glutamic acid (Glu), aspartic acid (Asp), *γ*-aminobutyric acid (GABA), and taurine (Tau) were obtained from Sigma (St. Louis, MO, USA). All other reagents used were of analytical grade.

### 2.4. Experimental Animals

Male Wistar rats weighing 180–220 g were purchased from the Laboratory Animal Center of Nanfang Medical University, Guangzhou, China. Animals were allowed 1 week to adapt to the surroundings before beginning any experimentation. Animals were maintained on a 12 h light/dark cycle under controlled temperature of 22 ± 2°C and had free access to food and water. All animals handling procedures were performed in strict accordance with the China legislation on the use and care of laboratory animals and were approved by the Animal Experimentation Ethics Committee of the Guangzhou University of Chinese Medicine. All efforts were made to minimize the number and suffering of animals needed to produce reliable data.

### 2.5. Chronic Unpredictable Mild Stress (CUMS)

**Figure 1:**
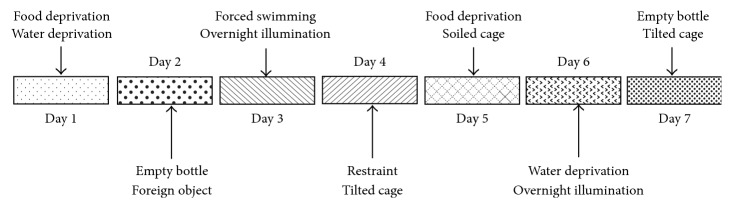
Schedule of chronic unpredictable mild stress (CUMS) procedure. The CUMS protocol consisted of the sequential application of a variety of unpredictable mild stressors. These stressors were randomly scheduled over a one-week period from day 1 to day 7 and repeated for 4 weeks during the entire experiment.

The stress procedure performed here mainly referred to Willner [6] with some modifications. Briefly, the CUMS protocol consisted of the sequential application of a variety of unpredictable mild stressors: (1) food deprivation for 24 hours, (2) water deprivation for 24 hours, (3) exposure to an empty bottle for 1 hour, (4) cage tilt (45°) for 7 hours, (5) overnight illumination, (6) soiled cage (200 mL water in 100 g sawdust bedding) for 24 hours, (7) forced swimming at 8°C for 6 minutes, (8) physical restraint for 2 hours, and (9) exposure to a foreign object (e.g., a piece of plastic) for 24 hours. These stressors were randomly scheduled over a one-week period and repeated throughout the 4 weeks of experiment (Figure 1). Nonstressed animals were left undisturbed in their home cages except during housekeeping procedures such as cage cleaning.

### 2.6. Treatment of Animals

In experiments, animals were randomized into seven groups of ten individuals. The control animals were unstressed and intragastrically given daily 2 mL distilled water. For another six groups, the animals were treated simultaneously with CUMS. The drugs (FA at 0.086 and 0.172 g/kg/day, FB at 1.804 and 3.608 g/kg, and IMI at 20 mg/kg/day, suspended in distilled water) were intragastrically given daily 1 h before the stress exposure for the entire 4 weeks. Following each stress session, animals were returned to their home cages and were able to access food and water freely for the remainder of the day.

### 2.7. Rat Sucrose Preference Test

Sucrose preference test was carried out at the end of 4-week CUMS exposure. The test was performed as described previously [7] with minor modifications. Briefly, after one week of the single cage feeding adaptation, the rats received a week of double bottle training. One bottle was 1% sucrose solution (w/v) 100 mL (in the cage on the left), another was pure water 100 mL (in the cage on the right), and two bottles were fixed. Sucrose solution and pure water were added every morning. After one-week training, the rats were deprived of water and food for 24 hours, and then 1 h sucrose solution consumption experiments were conducted to measure the basis of sucrose preference at 9:00 am in which rats were housed in individual cages and were free to access two bottles. After 4 weeks of experiment, the sucrose preference was conducted again by the method described above. The sucrose preference was calculated by the following formula: sucrose preference (%) = sucrose consumption/total liquid consumption × 100% [8].

### 2.8. Forced Swimming Test (FST)

The forced swimming test was performed according to the method of Porsolt [9] with modifications. Briefly, rats were forced to swim in a transparent glass vessel (25 cm in height and 14 cm in diameter) filled with 15 cm of water at 24 ± 1°C. The total duration of immobility (seconds) was measured during the last 4 minutes of a single 6-minute test session [10]. Rats were considered immobile when they ceased struggling and remained floating motionless in the water except the movements necessary to keep their heads above the water. Decrease in the duration of immobility during the FST was taken as a measure of antidepressant activity.

### 2.9. Tail Suspension Test (TST)

The duration of immobility time induced by tail suspension was measured according to the method of Steru [11]. Rats both acoustically and visually isolated were suspended above the floor by adhesive tape placed approximately 1 cm from the tip of the tail. The remaining immobile time of TST was quantified for 6 min. Rats were considered immobile only when they hung passively and completely motionless.

### 2.10. Open-Field Test (OFT)

The OFT was based on the method of Archer [12]. The OFT apparatus consisted of a clear acrylic box (100 cm × 100 cm × 50 cm) with a lid. The floor was divided by drawn lines into 36 areas of about 25 sq.cm. The test was performed in a light- and sound-attenuated shield box with a dim light. The locomotor activity counts and the rearing counts were recorded for 5 min. All test sessions were recorded by a video camera. Between subjects, the box was thoroughly cleaned with cotton and 95% ethanol.

### 2.11. CORT, CRH, ACTH, and AVP Contents in Plasma Assays

After the end of the experimental period, all rats were sacrificed by decapitation between 10:00 and 11:00 am in the laboratory. After decapitation, the blood samples were collected into heparinized Eppendorf tubes and immediately centrifuged for 1 min (10,000 rpm) at 4°C to separate plasma, which was then frozen at −20°C until subsequent assays. CORT, CRH, ACTH, and AVP contents in plasma were determined by using competitive radioimmunoassay according to the manufacturer's instruction.

### 2.12. CRH, ACTH, and AVP Contents in Hypothalamus Assays

After decapitation, the brains were rapidly removed and immersed into the boiling saline for 5 min. Hypothalamus was dissected according to the method described by Yan et al. [13], weighed, and then homogenized in 0.2 mL of 1 M acetic acid solution. After incubation for 100 minutes at room temperature, homogenate solution was mixed with 0.2 mL of 1 M NaOH solution and centrifuged for 20 min (3500 rpm) at 4°C. The supernatants were collected and stored at −40°C. Radioimmunoassay was performed to measure CRH, ACTH, and AVP in hypothalamus following the manufacturer's instruction.

### 2.13. Monoamine and Amino Acid Neurotransmitters Contents in Hippocampus Determination

After the end of the experimental period, all rats were sacrificed by decapitation between 10:00 and 11:00 am in the laboratory. After decapitation, the whole rat brains were rapidly removed and the hippocampus was carefully dissected according to the method described previously [14]. The contents of 5-HT, 5-hydroxyindoleacetic acid (5-HIAA), DA, and NE in hippocampus were determined by using an electrochemical high-performance liquid chromatography system (EC-HPLC). Tissues were placed in 0.1 N perchloric acid (including 0.1% aminothiopropionic acid), then sonicated, and centrifuged twice for 20 min at 10,000 rpm. The supernatants were stored at −80°C until being analyzed for the contents of DA, 5-HT, and NE. The pellets were digested in 1 mL of 0.5 N NaOH for measurements of protein concentration using Bio-Rad assay reagents. For EC-HPLC analysis, samples were loaded onto a Waters 7125 plus autosampler (Waters, Milford, USA) and the mobile phase was delivered at a constant rate of 1 mL/min by a Waters Model 515 pump (Waters, Milford, USA) through a C18, 5 mm, 250 mm × 4.6 mm analytical column (HiQsil, Japan) placed in a column heater (35°C). The LC amperometric potential was set to 0.75 V with reference to an Ag-AgCl reference electrode and the sensitivity of the detector was modified according to the content of amines in the sample. The mobile phase consisted of 0.1 M monosodium phosphate, 0.15 mM EDTA, 2 mM NaCl, 1.4 mM octyl sodium sulfate, and 12% methanol. The signal from the detector was recorded and the data was analyzed using a N2000 Workstation (Institute of Intelligent Information Engineering, Zhejiang University, Zhejiang, China). The monoamine neurotransmitter contents were expressed as ng/g weight of tissue.

Amino acids in hippocampus were determined by OPA precolumn derivatization and RP-HPLC with a high-performance liquid chromatography system (Hewlett-Packed 1100 series, USA). The derivatization procedure was accomplished by an online autoinjector. The derivatives were separated on a Hypersil ODS and the signals were detected with a programmable fluorospectrophotometer detector. Mobile phase A was 10 mmol/L, PH 7.2 sodium phosphate buffer (PB) containing 0.5% (*φ*) tetrahydrofuran, and B was PB-methanol-acetonitrile (50 : 35 : 15 by volume). The elution program was 100% A with 0% B at the start (0 min) and 0% A with 100% B at the end (25 min) of the program. OPA-reactive compounds were detected at excitation wavelength of 340 nm and emission wavelength of 450 nm, and chromatograms were analyzed.

### 2.14. Statistical Analysis

The data were expressed as mean ± standard deviation (SD). The significance of the difference was statistically evaluated using one-way analysis of variance (ANOVA) as well as the least significant difference test. *P* < 0.05 was considered statistically significant.

## 3. Results

### 3.1. Sucrose Preference Test in Each Group

**Figure 2:**
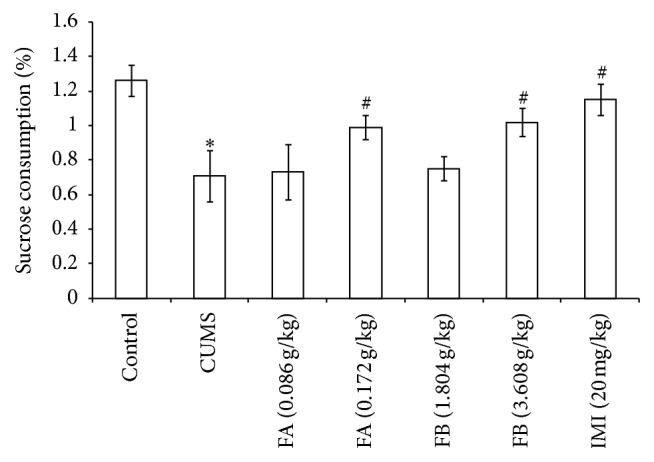
The effect of DZXYS (FA at 0.086 and 0.172 g/kg and FB at 1.804 and 3.608 g/kg, intragastrically) on the sucrose consumption in rats with chronic unpredictable mild stress. IMI at daily dosage of 20 mg/kg was set as a positive control. Each column represents mean ± SD, *n* = 10. ^*∗*^ *P* < 0.01 as compared with control group; ^#^ *P* < 0.01 as compared with CUMS group.

As shown in Figure 2, compared with the control group, CUMS decreased the sucrose preference in rats significantly (*P* < 0.01). Compared with the CUMS group, FA (0.172 g/kg), FB (3.608 g/kg), and IMI (20 mg/kg) significantly increased the sucrose preference (*P* < 0.01, Figure 2).

### 3.2. Forced Swimming Test (FST) in Each Group

**Figure 3:**
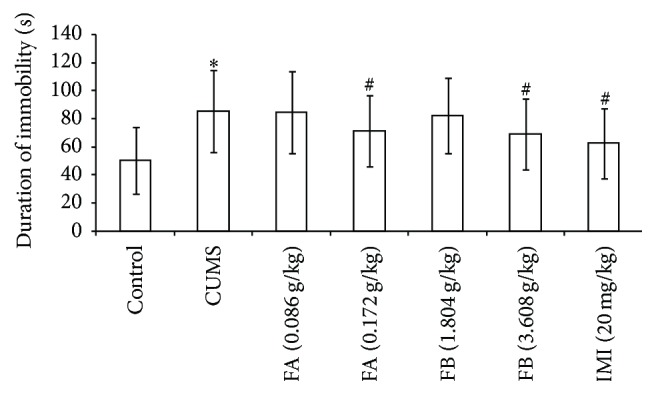
The effect of DZXYS (FA at 0.086 and 0.172 g/kg and FB at 1.804 and 3.608 g/kg, intragastrically) on the duration of immobility in FST in rats with chronic unpredictable mild stress. IMI at daily dosage of 20 mg/kg was set as a positive control. Each column represents the mean ± SD, *n* = 10. ^*∗*^ *P* < 0.05 as compared with control group; ^#^ *P* < 0.05 as compared with CUMS group.

In FST, compared with the control group, the duration of immobility in rats with CUMS increased significantly (*P* < 0.05). Compared with the CUMS group, FA (0.172 g/kg), FB (3.608 g/kg), and IMI (20 mg/kg) significantly decreased the duration of immobility (*P* < 0.05, Figure 3).

### 3.3. Tail Suspension Test (TST) in Each Group

**Figure 4:**
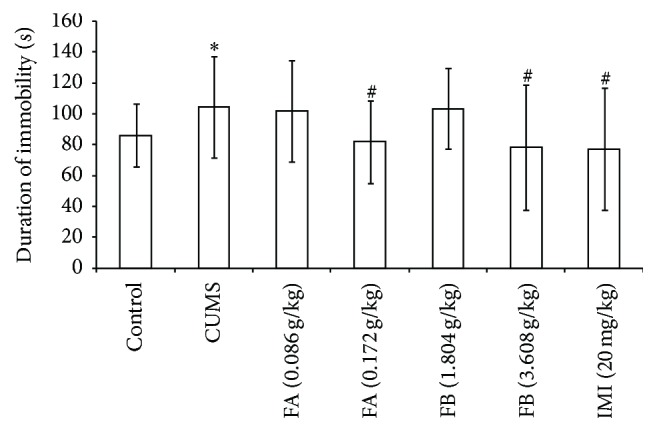
The effect of DZXYS (FA at 0.086 and 0.172 g/kg and FB at 1.804 and 3.608 g/kg, intragastrically) on the duration of immobility in TST in rats with chronic unpredictable mild stress. IMI at daily dosage of 20 mg/kg was set as a positive control. Each column represents the mean ± SD, *n* = 10. ^*∗*^ *P* < 0.01 as compared with control group; ^#^ *P* < 0.05 as compared with CUMS group.

In TST, compared with the control group, the duration of immobility in rats with CUMS increased significantly (*P* < 0.01). Compared with the CUMS group, FA (0.172 g/kg), FB (3.608 g/kg), and IMI (20 mg/kg) significantly decreased the duration of immobility (*P* < 0.05, Figure 4).

### 3.4. Open-Field Test (OFT) in Each Group

**Figure 5:**
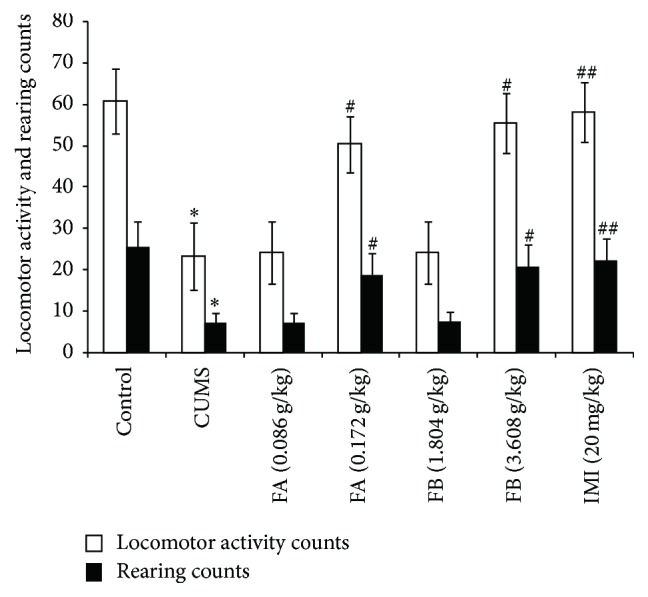
The effect of DZXYS (FA at 0.086 and 0.172 g/kg and FB at 1.804 and 3.608 g/kg, intragastrically) on the duration of immobility in OFT in rats with chronic unpredictable mild stress. IMI at daily dosage of 20 mg/kg was set as a positive control. Each column represents the mean ± SD, *n* = 10. ^*∗*^ *P* < 0.01 as compared with control group; ^#^ *P* < 0.05 and ^##^ *P* < 0.01 as compared with CUMS group.

In the OFT, compared with the control group, both the locomotor activity and the rearing counts for 5 min were lower in the rats with CUMS (*P* < 0.01). The results showed that the rats with CUMS appeared to have depressant-like behavioral changes. FA (0.172 g/kg), FB (3.608 g/kg), and IMI (20 mg/kg) significantly increased the locomotor activity and the rearing counts (*P* < 0.05 or *P* < 0.01, Figure 5).

### 3.5. CORT, CRH, and ACTH Contents in Plasma and Hypothalamus in Each Group

As shown in Table 2, CUMS significantly increased the contents of CORT, CRH, and ACTH in plasma in rats (*P* < 0.05). Compared with the control group, the contents of CRH and ACTH in hypothalamus in rats with CUMS were also higher (*P* < 0.05). Compared with the CUMS group, FA at 0.172 g/kg, FB at 3.608 g/kg, and IMI (20 mg/kg) significantly decreased the CORT, CRH, and ACTH contents in plasma and hypothalamus (*P* < 0.05 or *P* < 0.01).

### 3.6. AVP Contents in Plasma and Hypothalamus in Each Group

As shown in Table 3, CUMS significantly increased the AVP contents both in hypothalamus and in plasma (*P* < 0.01). The AVP contents could be significantly decreased in both hypothalamus and plasma by FA (0.172 g/kg), FB (3.608 g/kg), and IMI (20 mg/kg) (*P* < 0.05 or *P* < 0.01).

### 3.7. Monoamine Neurotransmitters Contents in Hippocampus in Each Group

**Figure 6:**
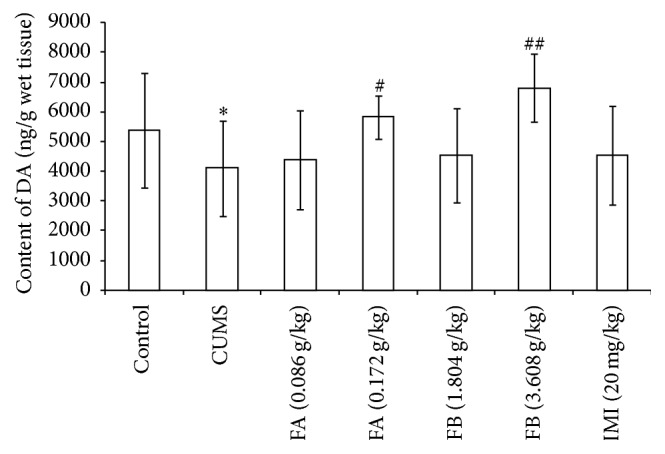
The effect of DZXYS (FA at 0.086 and 0.172 g/kg and FB at 1.804 and 3.608 g/kg, intragastrically) on the contents of DA in hippocampus in rats with chronic unpredictable mild stress. IMI at daily dosage of 20 mg/kg was set as a positive control. Each column represents the mean ± SD, *n* = 10. ^*∗*^ *P* < 0.01 as compared with control group; ^#^ *P* < 0.05 and ^##^ *P* < 0.01 as compared with CUMS group.

**Figure 7:**
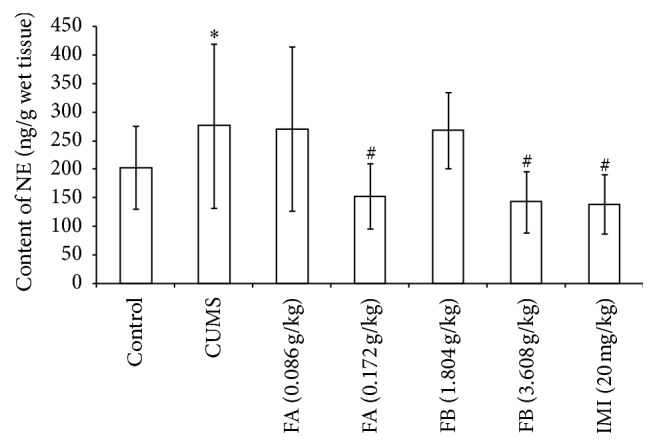
The effect of DZXYS (FA at 0.086 and 0.172 g/kg and FB at 1.804 and 3.608 g/kg, intragastrically) on the contents of NE in hippocampus in rats with chronic unpredictable mild stress. IMI at daily dosage of 20 mg/kg was set as a positive control. Each column represents the mean ± SD, *n* = 10. ^*∗*^ *P* < 0.01 as compared with control group; ^#^ *P* < 0.01 as compared with CUMS group.

As shown in Table 4 and Figures 6 and 7, compared with the control group, CUMS markedly decreased the contents of 5-HT and DA (*P* < 0.01) and increased the NE content in hippocampus (*P* < 0.01). FA (0.172 g/kg) and FB (3.608 g/kg) significantly increased the contents of 5-HT and DA and decreased the content of NE in hippocampus (*P* < 0.05 or *P* < 0.01). As a positive control, IMI (20 mg/kg) could increase the 5-HT content and decrease the NE content in hippocampus (*P* < 0.01) but had no effect on DA. As a metabolite of serotonin, the ratio of 5-HIAA/5-HT could be used as an index for the turnover of 5-HT [15, 16]. Here, the ratio of 5-HIAA/5-HT was calculated. The amount of 5-HIAA was not altered in hippocampus of rat with CUMS (Table 4). FB (3.608 g/kg) as well as IMI (20 mg/kg) significantly elevated the amount of 5-HIAA (*P* < 0.05). The ratio of 5-HIAA/5-HT (*P* < 0.05) was significantly increased by CUMS, which could be reversed by FA (0.172 g/kg), FB (3.608 g/kg), and IMI (20 mg/kg).

### 3.8. Amino Acid Neurotransmitters Contents in Hippocampus in Each Group

**Figure 8:**
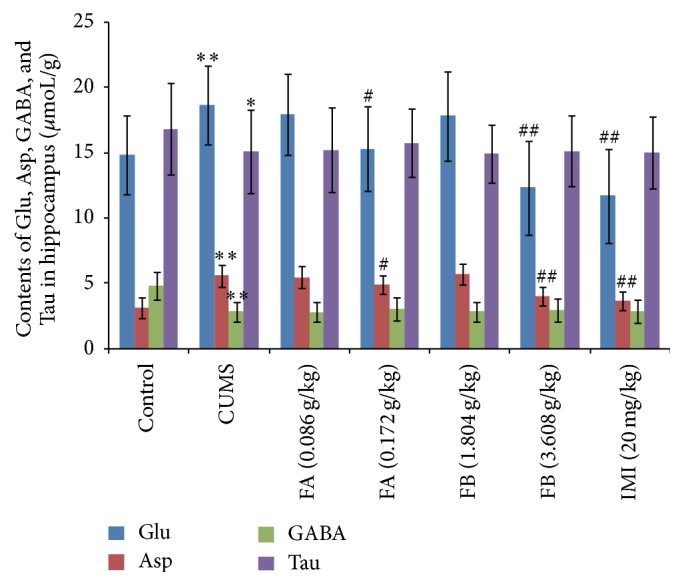
The effect of DZXYS (FA at 0.086 and 0.172 g/kg and FB at 1.804 and 3.608 g/kg, intragastrically) on the contents of Glu, Asp, GABA, and Tau in hippocampus in rats with chronic unpredictable mild stress. IMI at daily dosage of 20 mg/kg was set as a positive control. Each column represents the mean ± SD, *n* = 10. ^*∗*^ *P* < 0.05 and ^*∗∗*^ *P* < 0.01 as compared with control group; ^#^ *P* < 0.05 and ^##^ *P* < 0.01, as compared with CUMS group.

Compared with the control group, the contents of Glu and Asp in hippocampus were significantly higher (*P* < 0.01), while the GABA and Tau contents were significantly lower (*P* < 0.05 or *P* < 0.01). FA (0.172 g/kg), FB (3.608 g/kg), and IMI (20 mg/kg) significantly decreased the contents of Glu and Asp in the hippocampus (*P* < 0.05 or *P* < 0.01, Figure 8) but had no effects on GABA and Tau.

## 4. Discussion

Traditional Chinese medicine has offered a possible therapy for the treatment of depression. DZXYS, an ancient Chinese herbal decoction, has been used in Chinese medicinal herbal mixture for antidepression. However, its action mechanism has not been revealed. In the present study, we provided different lines of evidence to support the antidepression role of DZXYS in CUMS rat model system.

The pathogenesis of depression model established by using stress factors is more consistent with that of depression in human beings. The symptoms such as reduced activity, decreased exploratory behavior, and reduced sucrose preference appear in stressed animals, which are similar to the changes of behavior and the loss of interest or pleasure in the clinical diagnosis of depression [17]. The CUMS model of depression is accepted as a valuable method for evaluating antidepressant effects in animals. In the present study, FA (0.172 g/kg) and FB (3.608 g/kg) prepared from DZXYS exhibited a significant increase of sucrose preference in the rats with CUMS. The FST, TST, and OFT are the most widely used as behavioral tools for assessing antidepressant activity [18, 19]. Characteristic of rat behaviors scored in both tests is termed immobility, which reflects behavioral despair as seen in human depression. The duration of immobility time in rats that are trapped and forced to swim is closely related to helplessness [20]. It was observed from our results that there was significant prolongation of immobility time in these groups after modeling. The two fractions at 0.172 g/kg and 3.608 g/kg, respectively, could significantly decrease the duration of immobility both in FST and in TST and significantly increase the locomotor activity in OFT. These results may further confirm that the DZXYS has an antidepressant activity.

The nervous system and the endocrine system are closely related, which constitute a complete network to maintain homeostasis. HPA axis activation and the resulting increase in glucocorticoid (GC) secretion are the two most important features of the stress response. When in stress state, the hypothalamus releases CRH and AVP in response to a stressor, which in turn activate the secretion of ACTH from the pituitary, which finally stimulates the secretion of cortisol (in humans) or CORT (in rodents) from the adrenal cortex [21]. From the actual situation of human depression, stressful life events play an important role in depression. Stressor or stressful environment usually precedes depressive symptoms, which leads to neurobiochemical changes associated with depression [17]. Since the 1970s, people have found that the HPA axis is involved in the pathological mechanism of depression. Many studies suggested that HPA axis activation results in some of the symptoms and signs in depression [22]. Moreover, it has been found that the damage of HPA axis is more severe in patients with refractory depression [23]. Hyperactivity of HPA axis often occurs in both depressed patients and animal models, which is mainly presented as hypersecreted CRH and higher contents of ACTH and GC [24, 25]. Combined with receptors in multiple target tissues (including HPA axis), GC could exert physiological effects. When in state of stress, GC is hypersecreted and then combined with glucocorticoid receptor (GR), thereby inhibiting the hypothalamus to secrete CRF and AVP by negative feedback to restore the hyperactivated HPA axis to the basal level. However, chronic stress could result in the state of persistent hyperactivity of the HPA axis; excessive GC constantly stimulates GR, which leads to the injury of hippocampal neurons and the dysfunction of emotion regulation center, and then induces depression [26]. As observed in our experiment, the CUMS could significantly increase the contents of CORT, CRH, and ACTH in both plasma and hypothalamus in rats, accompanied by the depressive-like behavioral alterations; thus, we confirmed that HPA axis hyperactivity is related to the pathogenesis of depression. Results from the present study demonstrated that FA (0.172 g/kg) and FB (3.608 g/kg) prepared from DZXYS could significantly decrease the CORT, CRH, and ACTH contents in plasma and hypothalamus, which suggested that antidepressant effects exerted by DZXYS were partly due to inhibiting the hyperactivity of the HPA axis.

Some previous studies have shown that there are significant increases of AVP neurons expression and AVP level in the paraventricular nucleus, AVP reactivity in the pituitary, and AVP mRNA expression in the supraoptic nucleus in depressed patients [27, 28], which also suggests that AVP changes in the paraventricular nucleus and supraoptic nucleus may be the basis for the onset of depression. In addition, AVP level in the plasma in depressed patients is higher than that in the healthy control group, which is related to the severity of the disease [29]. AVP, a nonapeptide synthesized in hypothalamic nuclei, is critical for the regulation of the activity of the HPA axis representing a major component of the stress response. CRH and AVP act synergistically in bringing about ACTH release from the corticotropes of the anterior pituitary, which in turn stimulates cortisol output from the adrenal cortex. AVP is involved in the pathogenesis of depression by regulating the HPA axis function [30]. In order to further explore the effect of DZXYS on HPA axis, the levels of AVP in plasma and hypothalamus were measured in the present study. Our results showed that, in rats with CUMS, AVP levels significantly increased in plasma and hypothalamus, which could be significantly reversed by the treatment of FA (0.172 g/kg), FB (3.608 g/kg), and IMI (20 mg/kg). Combined with the results of HPA axis determination in our study, it suggested that DZXYS could regulate the HPA axis by acting on the AVP to exert antidepressant effect.

As a significant contribution to depression, “monoamine hypothesis” has been proposed in the 20th century, thereby setting up the foundation for today's antidepressant treatment. Monoamine neurotransmitters, such as 5-HT, DA, and NE, are the important bioactive substances in central nervous system which participate in many physiological activities of the body, including emotion, learning, and memory. NE plays an important role in maintaining arousal and is involved in the brain reward system and learning and memory function. The concentration of NE in the hypothalamus is significantly decreased, which suggests that the dysfunction of central NE is related to depression [31]. Dysfunction of 5-HT is related to depressed mood, anxiety, inhibition of movement, loss of appetite, sleep disorders, circadian rhythm disorders, and so on in depression [32]. Dopamine (DA) is involved in the regulation of motivation, volition, interest/pleasure, and attention/concentration, all of which are likely to be impaired in depressed patients. Several previous reports have suggested that depression may often be accompanied by a relative hypodopaminergic state [33]. According to the “monoamine hypothesis,” the decreased releasing and lower contents in the synapse of 5-HT, DA, or NE in central nervous system are involved in pathogenesis of depression. Thus, it is well known that many antidepressant drugs currently in use exert effects by regulating the reuptake and metabolic balance of monoamine neurotransmitters [34]. Our experimental results showed that the 5-HT and DA contents significantly decreased, while the NE content and the ratio of 5-HIAA/5-HT significantly increased in hippocampus in rats, accompanied by the depressive-like behavioral alterations, which confirm that the dysfunction of monoamine neurotransmitters plays an important role in the pathogenesis of depression.

Amino acid neurotransmitters can be divided into two types of excitatory amino acid and inhibitory amino acid; the former includes Glu and Asp, while GABA and Tau belong to the latter. Some of the studies, whether in rodent models of depression or in depressed patients, indicate that the increase of the Glu concentration is closely related to depression [35]. As an important inhibitory neurotransmitter in the brain, GABA decreases significantly in the patients of depression [36]. Therefore, the change of the ratio of excitatory/inhibitory amino acid in the brain has an important influence on the incidence of depression [37]. In the present study, we found that the contents of Glu and Asp significantly increased, while the GABA and Tau contents decreased in hippocampus in depressed rats; it is suggested that CUMS could lead to the imbalance of excitatory/inhibitory amino acids in the brain, which may account for the mechanism underlying the behavioral changes in rats.

In the rat and other mammalian species, including human beings, HPA axis and neurotransmitters system are closely interacted in central nervous system (CNS) (particularly in hippocampus) and greatly involved in stress related disorder [38, 39]. Under the chronic stress, as high concentration of GC could induce the liver to produce tryptophan pyrrole enzyme, it could degrade the tryptophan in the blood. Tryptophan is the precursor of 5-HT and its reduction could lead to synthesis of 5-HT and so lead to 5-HT content decrease in the brain, thus causing depressive symptoms [40]. In a stress state, HPA axis function becomes hyperactive and 5-HT synthesis significantly decreases as a result of insufficient tryptophan transported into the CNS [41]. Moreover, high concentration of GC can make 5-HT transporter (5-HTT) expression increase in hippocampus, amygdala, dorsal raphe nucleus, and other brain areas by GR dependent manner. The increased 5-HTT will increase the reuptake of 5-HT in synaptic cleft, which aggravates the decrease of 5-HT concentration and the severity of the symptoms in depressed patients [42]. Stress can affect the excitability of neurons of locus coeruleus (LC), raphe nucleus, and substantia nigra by HPA axis and 5-HT and NE/DA play an important role in the change of emotion and mood [43]. In the study of social stress in mice, by using the intracellular recording technique in vitro, it is found that CRH can directly activate noradrenergic neurons of the LC, thus resulting in the increase of NE in the brain [44]. In addition, some of the studies indicate that 5-HT, DA, and NE can stimulate the hypothalamus to secret CRH, which suggest that a positive feedback neural circuit exists between hypothalamic neurons and neurons secreting 5-HT, DA, and NE [45]. In chronic stress, high GC concentration may increase the releasing of excitatory amino acid from the synaptic vesicle of the glutamatergic nerve endings and inhibit its reuptake at the same time, which causes the persistent increase of excitatory amino acid [46]. The accumulation of Glu can cause hippocampal toxicity, and the high level of extracellular Glu may in turn make the HPA axis more hyperactive to secrete more GC [47]. Glu directly injected into paraventricular nucleus (PVN) in hypothalamus could accelerate the release of ACTH [48]. GABA could inhibit the releasing of ACTH, CORT, and CRH in hypothalamus and reregulate the hyperactivity of HPA axis; Tau, as an inhibitory amino acid, has the synergistic action with GABA to exert the effect on inhibiting the activation of HPA axis [49, 50].

Our data indicated that FA (0.172 g/kg) and FB (3.608 g/kg) significantly increased the 5-HT and DA contents and decreased the NE content in the hippocampus; meanwhile, the ratio of 5-HIAA/5-HT in rats with CUMS could be reversed by FA (0.172 g/kg) and FB (3.608 g/kg), which may account for primary monoamine neurotransmitters regulating mechanism underlying DZXYS antidepressant effects. In the present study, we found that although having no effects on GABA and Tau, FA (0.172 g/kg) and FB (3.608 g/kg) significantly decreased the contents of Glu and Asp in hippocampus, which suggested that DZXYS could reduce the excitatory toxicity caused by the accumulation of Glu and Asp in hippocampus in depressed rats, partly regulating the imbalance of the ratio of excitatory/inhibitory amino acid that may be involved in its antidepressant effects. There is a close relationship between HAP and neurotransmitters in regulating emotion and behavior in depression [51]. As observed in our experiment, it could be speculated that the amelioration produced by DZXYS on the neurotransmitter system may be associated with its effect on HPA axis. However, the cellular and molecular biological mechanism of exerting regulating effect of DZXYS between the HPA axis and neurotransmitters remains to be further studied in the future.

In conclusion, in the present study, the antidepressant effects of two fractions (petroleum ether soluble fraction, FA, and water-EtOH soluble fraction, FB) prepared from DZXYS were observed. The results demonstrated that DZXYS could ameliorate the depression-like behavior in chronic stress model of rats. The mechanisms of action of DZXYS might be accounted for by inhibiting hyperactivity of HPA axis and modulating monoamine and amino acid neurotransmitters in the hippocampus. Thus, we confirmed that DZXYS has the potential to be a beneficial remedy for the treatment of depression. Because of multiple using of plants in a traditional herbal medicine and the complexity of the pathological mechanism of depression, it is undoubted that there exist more other active fractions from DZXYS and further regulatory mechanisms of DZXYS in antidepression. These works are currently being conducted in our laboratory.

## Figures and Tables

**Table 1:** Different components in the formula of DZXYS.

Pharmaceutical name	Family name	Plant part	Chinese name
Radix Bupleuri	Umbelliferae	Root	Chai hu
Paeoniae Radix Alba	Ranunculaceae	Root	Bai shao
*Angelica sinensis*	Umbelliferae	Root	Dang gui
Bighead atractylodes rhizome	Compositae	Rhizoma	Bai zhu
*Poria cocos*	Polyporaceae	Sclerotium	Fu ling
Bark of tree peony root	Ranunculaceae	Root-bark	Mu dan pi
Fructus Gardeniae	Rubiaceae	Fruit	Zhi zi
*Mentha haplocalyx*	Lamiaceae	Leaf	Bo he
Licorice	Leguminosae	Root	Gan cao

**Table 2:** The contents of CORT, CRH, and ACTH in plasma and hypothalamus in each group.

Group	Dose	CORT	CRH		ACTH	
		Plasma (ng/mL)	Plasma (ng/mL)	Hypothalamus (ng/g)	Plasma (ng/mL)	Hypothalamus (ng/g)
Control	—	12.60 ± 6.88	3.31 ± 0.64	20.65 ± 12.52	42.11 ± 19.55	19.65 ± 8.97
CUMS	—	22.69 ± 13.49^*∗*^	7.38 ± 1.78^*∗*^	36.74 ± 8.73^*∗*^	51.44 ± 25.80^*∗*^	22.79 ± 7.33^*∗*^
FA (g/kg)	0.086	22.32 ± 13.31	7.37 ± 1.63	35.35 ± 8.02	50.56 ± 24.71	22.42 ± 7.32
	0.172	14.74 ± 10.30^##^	5.33 ± 0.79^#^	23.02 ± 12.8^##^	46.83 ± 23.32^##^	20.83 ± 6.92^#^
FB (g/kg)	1.804	20.00 ± 6.71	7.25 ± 1.77	33.36 ± 10.57	51.32 ± 10.28	22.26 ± 9.20
	3.608	10.91 ± 3.30^##^	3.95 ± 0.12^##^	16.04 ± 9.34^##^	44.47 ± 20.31^#^	20.59 ± 6.88^#^
IMI (mg/kg)	20	10.23 ± 3.21^##^	3.67 ± 0.13^##^	15.44 ± 9.02^##^	42.26 ± 20.01^##^	19.13 ± 6.76^##^

Values are expressed as mean ± SD (*n* = 10). ^*∗*^ *P* < 0.05, as compared with control group. ^#^ *P* < 0.05 and ^##^ *P* < 0.01, as compared with CUMS group.

**Table 3:** The AVP contents in plasma and hypothalamus in each group.

Group	Dose	AVP	
		Plasma (pg/mL)	Hypothalamus (pg/mg)
Control	—	14.70 ± 4.80	58.50 ± 17.80
CUMS	—	25.20 ± 7.30^*∗*^	309.20 ± 70.41^*∗*^
FA (g/kg)	0.086	24.23 ± 7.12	306.20 ± 70.33
	0.172	12.60 ± 3.20^#^	267.40 ± 58.60^#^
FB (g/kg)	1.804	23.74 ± 7.10	301.60 ± 70.70
	3.608	12.30 ± 3.40^##^	173.20 ± 20.90^##^
IMI (mg/kg)	20	11.21 ± 3.12^##^	168.53 ± 20.11^##^

Values are expressed as mean ± SD (*n* = 10). ^*∗*^ *P* < 0.01, as compared with control group. ^#^ *P* < 0.05 and ^##^ *P* < 0.01, as compared with CUMS group.

**Table 4:** The contents of 5-HT, 5-HIAA, and 5-HIAA/5-HT ratio in hippocampus in each group.

Group	Dose	5-HT (ng/g wet tissue)	5-HIAA (ng/g wet tissue)	5-HIAA/5-HT
Control	—	428.3 ± 171.2	376.3 ± 210.3	1.04 ± 0.41
CUMS	—	213.7 ± 142.4^*∗∗*^	387.6 ± 146.6	1.62 ± 0.82^*∗*^
FA (g/kg)	0.086	262.5 ± 145.8	370.6 ± 107.8	1.58 ± 0.83
	0.172	447.8 ± 236.7^#^	436.8 ± 311.2	1.02 ± 0.46^#^
FB (g/kg)	1.804	301.2 ± 146.5	378.7 ± 118.1	1.57 ± 0.79
	3.608	468.2 ± 139.3^##^	487.2 ± 83.2^#^	0.97 ± 0.56^#^
IMI (mg/kg)	20	479.5 ± 148.7^##^	509.3 ± 84.7^#^	1.01 ± 0.33^#^

Values are expressed as mean ± SD (*n* = 10). ^*∗*^ *P* < 0.05 and ^*∗∗*^ *P* < 0.01, as compared with control group. ^#^ *P* < 0.05 and ^##^ *P* < 0.01, as compared with CUMS group.
